# Open Reduction and External Fixation of a Comminuted Intra-articular Fifth Metacarpal Head Fracture: A Case Report

**DOI:** 10.7759/cureus.38845

**Published:** 2023-05-10

**Authors:** David Seaton, Gur Sidhu, Christos Kitsis, Neil Ashwood

**Affiliations:** 1 Trauma and Orthopaedics, University of Leicester Medical School, Leicester, GBR; 2 Trauma and Orthopaedics, University Hospitals of Derby and Burton NHS Foundation Trust, Derby, GBR; 3 Trauma and Orthopaedics, Wolverhampton University Research Institute, Wolverhampton, GBR

**Keywords:** metacarpophalangeal joint involvement, external fixation, comminuted, intra-articular, metacarpal head fracture

## Abstract

Comminuted intra-articular fractures are among the most difficult to fix, with open reduction and internal fixation often being impossible. We report the case of a 15-year-old male who required an open reduction with external fixation after sustaining an extremely comminuted intra-articular fifth metacarpal head fracture of the right hand. The patient presented with swelling localised to the fourth and fifth dorsal metacarpals of the right hand, with radiographs demonstrating an intra-articular fracture with comminution and articular surface depression.

Literature surrounding metacarpal head fractures, although scarce, suggests that whilst treatment must be individualised, most osteochondral fractures can be managed via open reduction with internal fixation either via K wires, interfragmentary screws or small headless screws. This case demonstrates that in challenging cases, with limited bone stock and cavities created through reduction of the fracture, fixation can be achieved through K wire with HK2 external fixation. It also highlights the apparent insufficiency in articles specifically detailing potential management options for intra-articular metacarpal fractures and has provided evidence of one potential fixation method.

## Introduction

Metacarpal head injuries are usually caused by direct trauma, often resulting in comminuted intra-articular or peri-articular fractures. This is a similar mechanism to metacarpal shaft and neck fractures however these can also result due to torsional and axial loading, associated with a closed or clenched fist [1]. The highest rate of incidence with respect to metacarpal fractures is in men aged between 10 and 29 [2], with some literature reporting that metacarpal fractures account for 40% of all hand fractures [3].

Patients often present with bruising, swelling and diffuse pain over the dorsal aspect of the hand, potentially associated with a loss of knuckle prominence and rotational deformity; no degree of malrotation is acceptable [4]. Radiographs are often first-line imaging, with recommended views being posteroanterior, lateral and ER oblique; with the Brewerton view helping to visualise collateral avulsion fractures [5]. Alternative diagnoses include fractures of the neck, shaft, base [3] and dislocations of the metacarpophalangeal joint.

This article was previously presented as a poster at the 2022 British Association of Clinical Anatomists Winter Scientific Meeting on 20^th^ December 2022 in Liverpool, England.

## Case presentation

**Figure 1:**
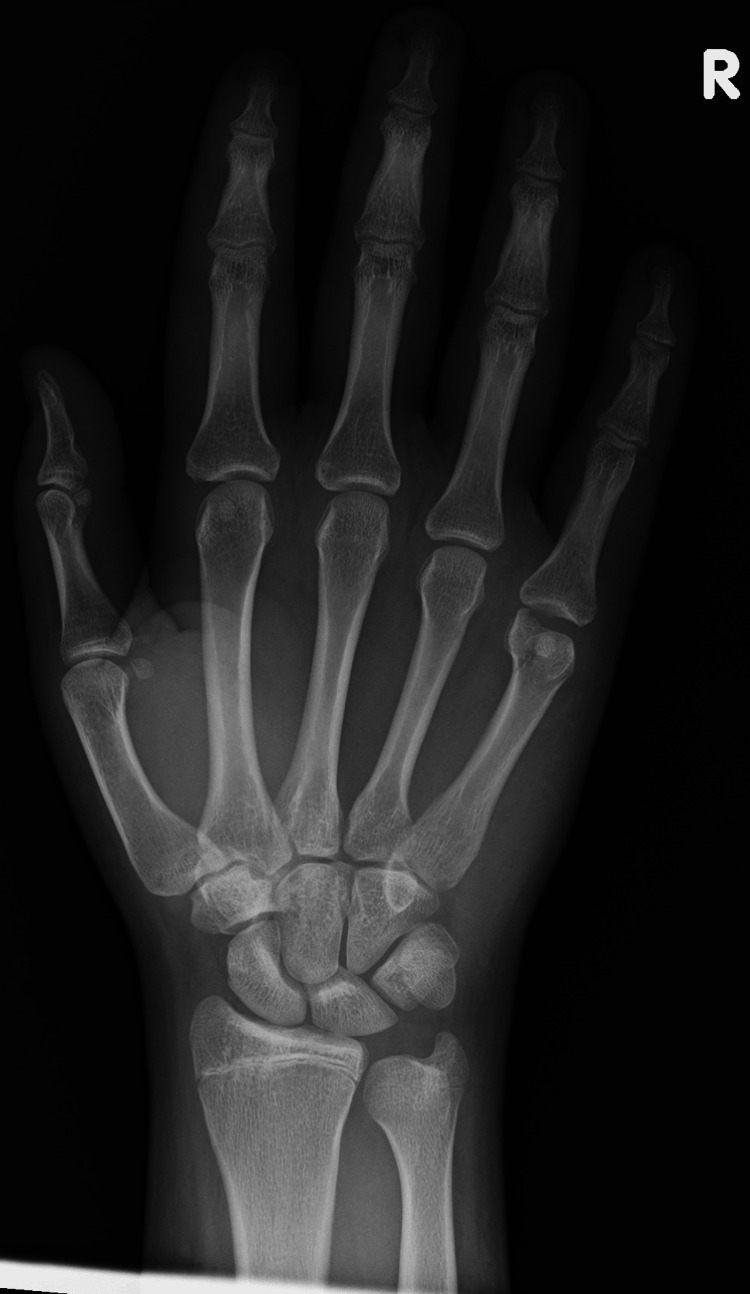
The patient's posteroanterior radiograph taken on presentation to the emergency department.

**Figure 2:**
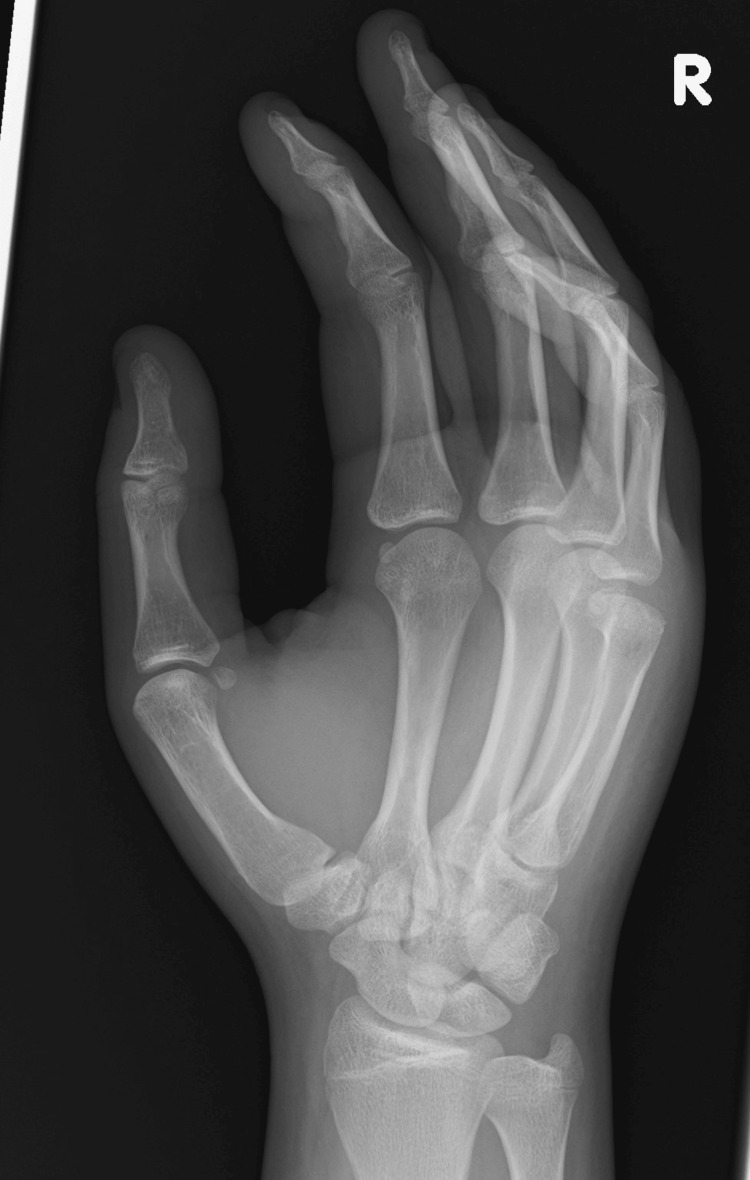
The patient's oblique radiograph taken on presentation to the emergency department.

A 15-year-old male presented with pain in the right hand following a physical altercation at school. Initially self-managed, with no improvement after two days the patient subsequently presented to the emergency department with pain and swelling over the dorsal aspect of the fourth and fifth metacarpals, however no erythema, angular deformity or external wounds were observed. The patient participated in boxing and was concerned that they were unable to at the time of their presentation. Right hand radiographs, as seen in Figures 1, 2, demonstrated an intra-articular fracture to the fifth metacarpal head with comminution and depression of the articular surface.

**Figure 3:**
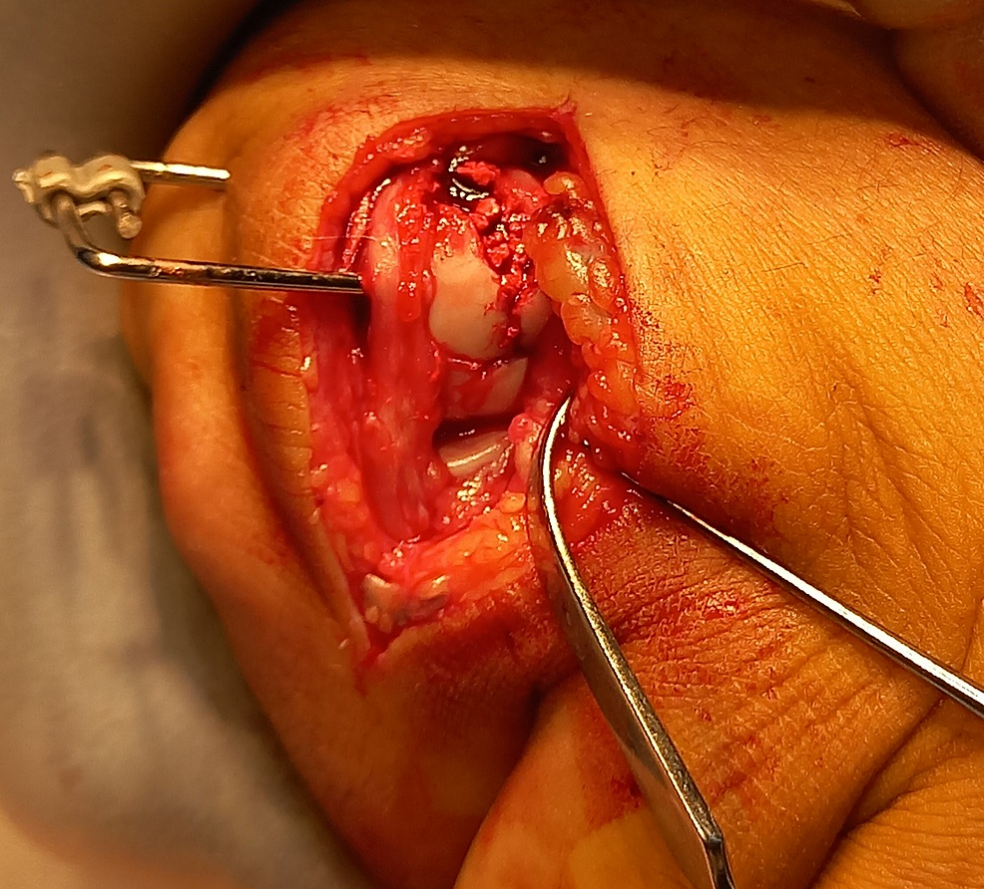
The fixation device in situ, prior to closure of the incision in theatre.

**Figure 4:**
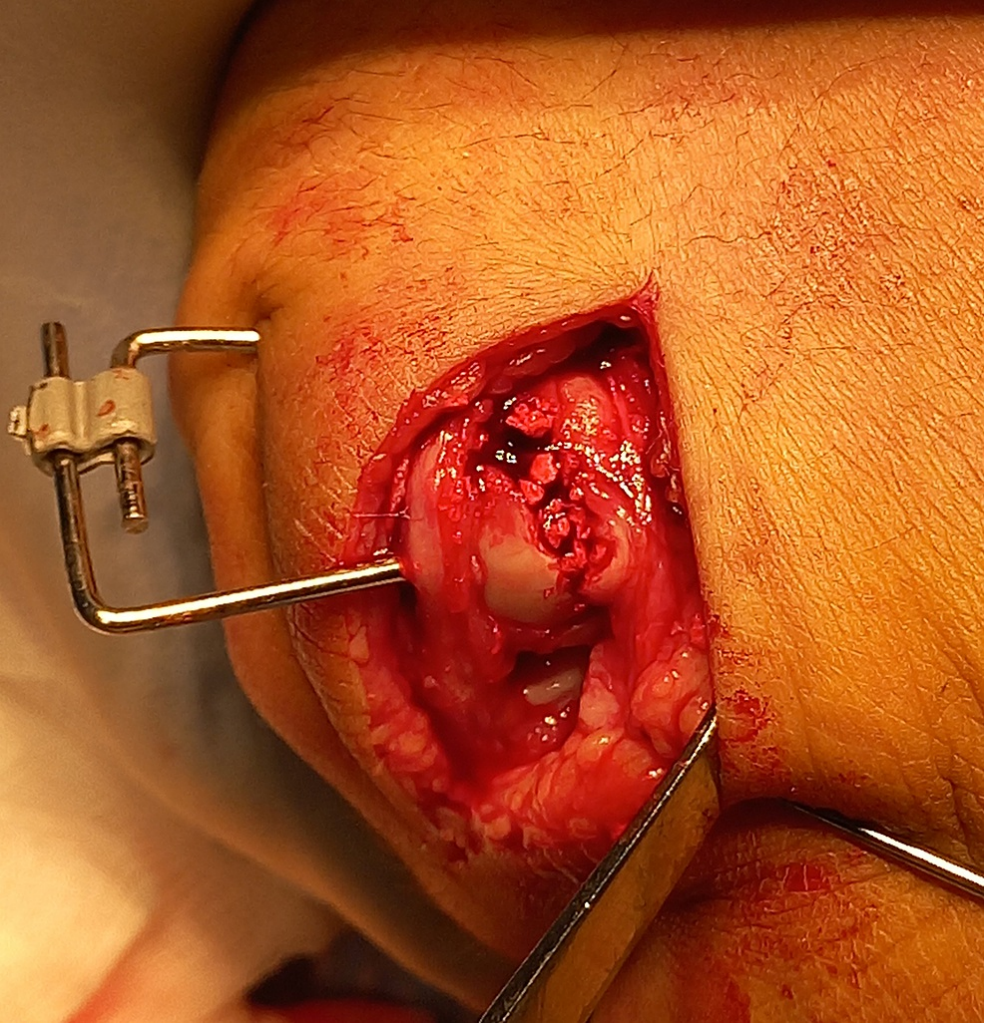
The fixation device in situ, prior to closure of the incision in theatre.

**Figure 5:**
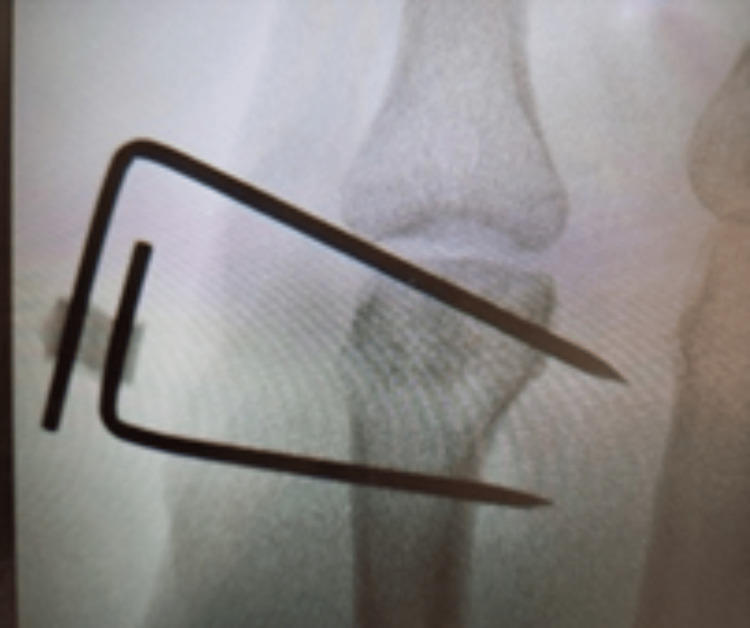
An intra-operative radiograph detailing the fixation device.

During the fracture clinic follow-up appointment, both conservative and surgical treatment options were discussed including the risks and benefits of specific options with the patient. The main fragment had a 3-4mm impacted depression creating a cavity with a subsequent loss of bone stock, meaning a headless screw fixation was not viable. Stable fracture fixation was subsequently achieved with an HK fixator (AREX, Palaiseau, France), as can be seen in Figures 3-5.

**Figure 6:**
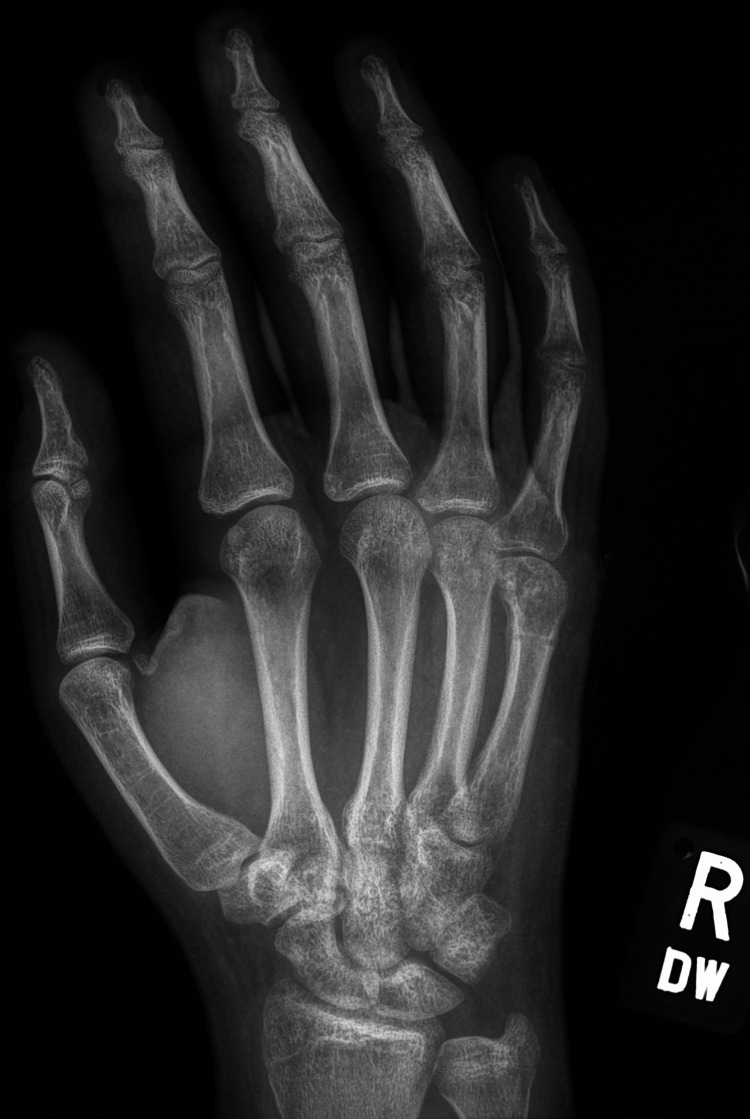
The patient's posteroanterior radiograph immediately following removal of the fixator.

**Figure 7:**
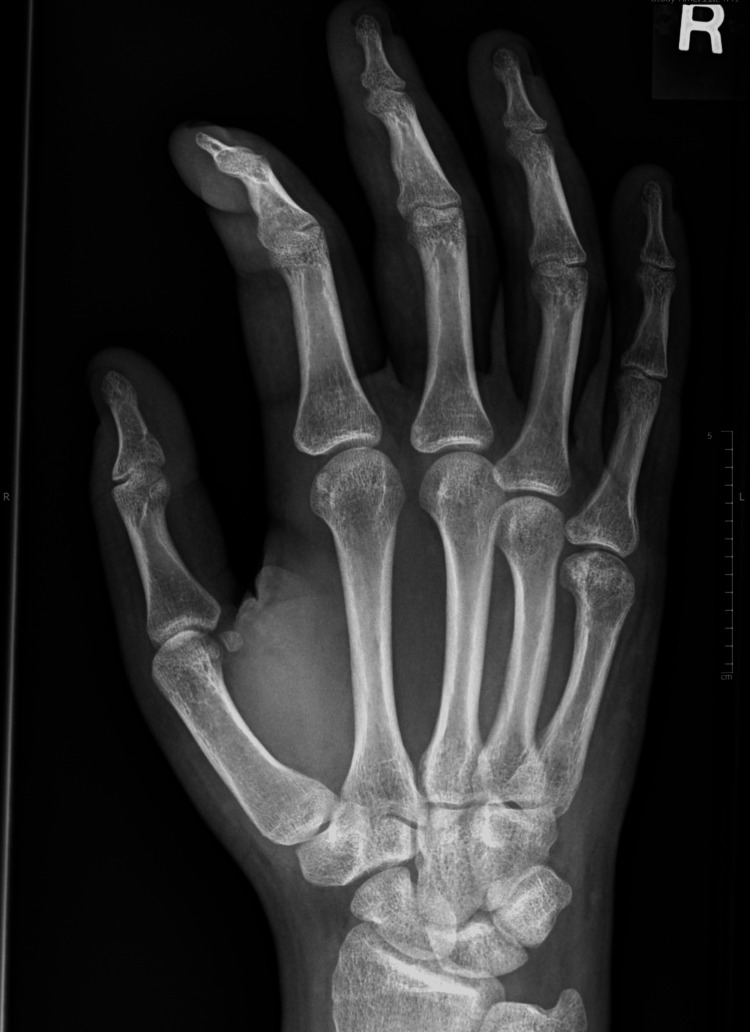
The patient's radiograph taken 12 weeks following removal of the fixator.

As this is a recent case, an accurate conclusion cannot yet be obtained as to the patient’s overall recovery. Despite this the patient reported that they were progressing well with minimal stiffness at an appointment to remove the fixator. Radiographs following the removal of the fixator can be seen in Figures 6, 7. It is worth noting that retrospective consent was gained from the patient’s parents immediately following the operation as the complex nature of the fixation only became apparent during the surgery.

## Discussion

A Boxer’s fracture is defined as a fracture of the fifth metacarpal neck, with the distal fragment displaced anteriorly [6], and accounts for around 10% of all hand fractures [3]. It is important to differentiate the diagnosis and treatment of metacarpal neck fractures with those of metacarpal head fractures.

Metacarpal fracture treatment depends on the extent of the injury with location, comminution, angulation, and rotational deformities being significant factors in this decision. Posteroanterior, lateral and oblique radiograph views must be obtained to evaluate the extent of a metacarpal head injury, however for injuries following a clenched fist, the Skyline metacarpal view may help visualise the dorsal articular surface [7]. Whilst literature notes that the use of Computerised Tomography may enhance understanding and assessment for comminuted, intraarticular involvement [8], it is not commonly used for the diagnosis of metacarpal injuries [9]. For distal radial fractures, particularly with senior hand surgeons, it was found that intra-observer reproducibility did not improve alongside the introduction of Computerised Tomography imaging when classifying radiographs of various patients [10].

Non-operative management of metacarpal head fractures is reported by Gaston to revolve around immobilisation for two weeks, with flexion of the metacarpophalangeal joint at 70 degrees, followed by joint exercises [6]. Movement of joints is important to prevent shortening of the collateral ligaments and a subsequent loss of range of motion and functional impairment [3].

Surgical intervention is indicated with unacceptable amounts of angulation, any degree of rotation, instability, 2-3mm shortening, 25% articular involvement or more than 1mm articular step off [11]. Options can include open reduction and internal fixation of displaced a metacarpal head, and closed reduction or traction to manage a comminuted metacarpal head [12].

It has been suggested that intramedullary screw fixation can provide a suitable alternative to percutaneous k-wire and plate fixation, with a double-screw construct helping to prevent metacarpal shortening in the case of comminuted subcapital fractures [13]. The use of headless screws is supported due to the improved stability rotationally, and the lack of need for removal, especially when compared to fixation with K-wires [14], although only in the presence of metacarpal neck fractures [15]. However, Guidi et al. also state that the technique should not be used for subchondral fractures as there is a lack of purchase for the screw head [13].

Literature surrounding metacarpal head fractures suggests that whilst treatment must be individualised, most osteochondral fractures can be managed via open reduction with internal fixation either via K wires, interfragmentary screws or small headless screws [16]. Osteochondral fragments of smaller scale should not be individually fixed internally, but rather secured in place by fixation of larger fragments [17]. Gaston notes that comminuted articular fractures are among the most difficult to fix, with open reduction and internal fixation often impossible [6].

Most authors detailing intra-articular metacarpal fractures of the metacarpal base demonstrated report that fractures of the second and third metacarpal base should be treated with open reduction and stabilisation with K wires [18]. Although this is not directly comparable to this specific case, it is worth noting as it details fixation of fractures involving a joint surface within the hand.

## Conclusions

Literature surrounding metacarpal head fractures, although scarce, suggests that whilst treatment must be individualised, most osteochondral fractures can be managed via open reduction with internal fixation either via K wires, interfragmentary screws or small headless screws. This case demonstrates that in challenging cases, with limited bone stock and cavities created through reduction of the fracture, fixation can be achieved through K wire with external fixation via HK2. It also highlights the apparent insufficiency in articles specifically detailing potential management options for intra-articular metacarpal fractures, and has provided evidence of one potential fixation method.
